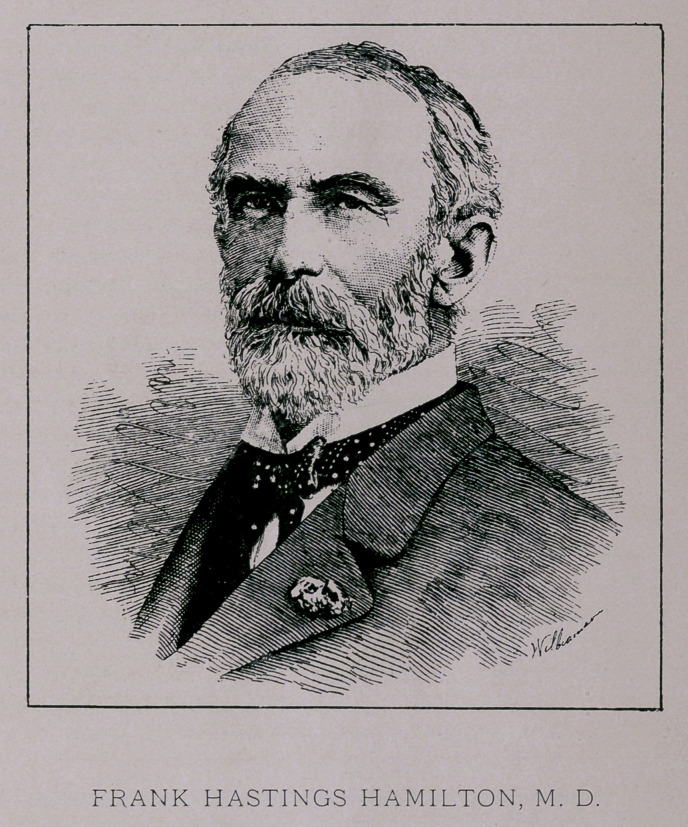# Frank Hastings Hamilton, M. D., LL. D.

**Published:** 1886-09

**Authors:** 


### Editorial.

### *FRANK HASTINGS HAMILTON, M. D., LL. D.*

On Monday, Aug. 11th, there died, in New York, the greatest surgeon that city had ever known, Dr. Frank H. Hamilton. He was great in knowledge, great in reputation and great in heart. While possessing such a superior knowledge, he was always modest and listened attentively to instruction from those qualified to impart, but with the imposter and charlatan he had no patience. It is a strange coincidence that the two great men —Flint and Hamilton—who have labored a life time together, and whose names were ever associated, should have departed this life at about the same time. Dr. Hamilton had one marked characteristic—candor and fair treatment of professional brethren —ever ready to defend or assist a physician in trouble, or when unjustly accused of error. To the profession he was best known through his book on "Fractures and Dislocations;" to the general public, through his connection with the Garfield case Besides his works on surgery, which had just been revised and re-published, he was ever before the medical public in articles in the various medical journals. One of his latest contributions was an article in this journal, on "Defence of Army Surgeons during he War." But to be noticed in the public press, either favorably or otherwise, was very distasteful to him. He was greatly annoyed at the constant mention of his name in connection with the Garfield case. He died beloved and renowned as few are.

We are indebted to the Buffalo *Morning Express* for the cut which we print herewith.

"Dr. Hamilton was born in Wilmington, Vt., in 1813, was graduated from the medical department of the University of Pennsylvania in 1833, and first settled in Auburn, N. Y. He was a professor in surgery at the Fairfield Medical School in 1839,

FRANK HASTINGS HAMILTON, M. D.

and in the following year at the Geneva Medical College. Removing from Auburn to Buffalo, in 1849, he became associated, two years later, with the late Dr. Austin Flint and Dr. James Platt White, in establishing the Medical Department of the University of Buffalo. There he taught his favorite branch—surgery.

"He removed to Brooklyn in 1860 and was the first professor of surgery in the Long Island Hospital. Early in the Civil War, in 1861, Dr. Hamilton became surgeon of the 36th New York Regiment, and was made Brigade Surgeon after the battle of Bull Run, and surgeon of Gen. Keyes' corps in 1862. A year later he became a medical inspector of the United States Army, in which position his work was notable.

"Of the Bellevue Hospital Medical College he was one of the founders in 1861, and professor of surgery there from that time to his resignation in 1875. As a writer on medical subjects, Dr. Hamilton was voluminous, learned and an accepted authority.

"Dr. Hamilton, in December, 1885, had severe and repeated pulmonary hemorrhages, due to fibroid phthisis, and since then has been failing in health. He has not been a robust man, but possessed wonderful vitality and will-power, which enabled him to do a tremendous amount of work. Within the past two or three weeks he began to grow very feeble. He suffered from innutrition, with no advance in his pulmonary trouble. This gradually sapped his vitality, and his weakness rapidly increased. His mental faculties, however, remained remarkably clear, and he had a complete understanding of his own ailment and its inevitable fatal ending. His own case he discussed, with his attending physicans, in a calm, impersonal way."

## Figures and Tables

**Figure f1:**